# The CROWN Initiative: journal editors invite researchers to develop core outcomes in women’s health

**DOI:** 10.1186/2054-7099-1-8

**Published:** 2015-06-02

**Authors:** Khalid Khan, on behalf of Chief Editors of Journals participating in The CROWN Initiative listed at the end of this article

**Affiliations:** BJOG: An International Journal of Obstetrics and Gynaecology, 27 Sussex Place, NW1 4RG London, UK

**Keywords:** Research design/standards, treatment outcome, endpoint determination/standards, clinical trials, systematic reviews, guidelines, bias (Epidemiology), evidence-based medicine, consensus

Clinical trials, systematic reviews and guidelines compare beneficial and non-beneficial outcomes following interventions. Often, however, various studies on a particular topic do not address the same outcomes, making it difficult to draw clinically useful conclusions when a group of studies is looked at as a whole [1]. This problem was recently thrown into sharp focus by a systematic review of interventions for preterm birth prevention, which found that among 103 randomised trials, no fewer than 72 different outcomes were reported [2]. There is a growing recognition among clinical researchers that this variability undermines consistent synthesis of the evidence, and that what is needed is an agreed standardised collection of outcomes – a “core outcomes set” – for all trials in a specific clinical area [1]. Recognising that the current inconsistency is a serious hindrance to progress in our specialty, the editors of over 50 journals related to women’s health have come together to support The CROWN (CoRe Outcomes in WomeN’s health) Initiative (Figure 1).

**Figure 1:**
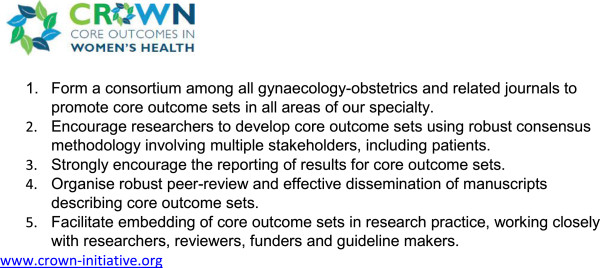
**Aims of The CROWN Initiative.**

Development of consensus is required around a set of well-defined, relevant and feasible outcomes for all trials concerning particular obstetric and gynaecologic health conditions, such as preterm birth, incontinence, infertility and menstrual problems. With so many subspecialties involved, this is no easy task. Duplication of effort can be avoided by working with the Core Outcome Measures in Effectiveness Trials (COMET) Initiative, which is working towards core data sets for all medical specialties [3]. Production of trustworthy core outcome sets will require engagement with patients, healthcare professionals, researchers, industry and regulators, and the employment of scientifically robust consensus methods [1]. The data for these core outcome sets, once agreed upon, should be collected in trials and reported in publications as standard practice in the future.

Journal editors now invite researchers to take the lead in beginning this work. What will we do as editors to support them and their colleagues? First, we are drawing wide attention to The CROWN Initiative by publishing this editorial in the journals listed below. We shall ensure that the global research community, which includes our many reviewers, is aware of the need for core outcome sets. Submissions which describe development of core outcome sets, if deemed acceptable after peer review, will be effectively disseminated.

Our collaboration is not for enforcing harmony at the expense of innovation. To quote from the COMET home page (http://www.comet-initiative.org): “The existence or use of a core outcome set does not imply that outcomes in a particular trial should be restricted to those in the relevant core outcome set. Rather, there is an expectation that the core outcomes will be collected and reported, making it easier for the results of trials to be compared, contrasted and combined as appropriate; while researchers continue to explore other outcomes as well.” We also expect that as new or superior ways of capturing outcomes emerge, core outcome sets will themselves need updating.

Producing, disseminating and implementing core outcome sets will ensure that critical and important outcomes with good measurement properties are incorporated and reported. We believe this is the next important step in advancing the usefulness of research, in informing readers, including guideline and policy developers, who are involved in decision-making, and in improving evidence-based practice.

## Note

Reproduced from ***The Core Outcomes in Women’s Health (CROWN) Initiative*** by Professor Khalid Khan with permission from the Royal College of Obstetricians and Gynaecologists and John Wiley & Sons Ltd.

## Appendix 1

The CROWN Initiative includes the following journals, in alphabetical order (correct on 13^th^ May 2014, up to date list available at http://www.crown-initiative.org):

1. Acta Obstetricia et Gynecologica Scandinavica
2. American Journal of Obstetrics & Gynecology
3. American Journal of Perinatology
4. Archives of Gynecology and Obstetrics
5. Australian and New Zealand Journal of Obstetrics and Gynaecology
6. Best Practice & Research: Clinical Obstetrics & Gynaecology
7. Birth: Issues in Perinatal Care
8. BJOG: An International Journal of Obstetrics and Gynaecology
9. BMC Pregnancy and Childbirth
10. BMC Women's Health
11. Climacteric
12. Clinical Obstetrics and Gynecology
13. Clinics in Perinatology
14. Cochrane Menstrual Disorders and Subfertility Group
15. Cochrane Pregnancy and Childbirth Group
16. Contraception
17. Contraception and Reproductive Medicine
18. Current Opinion in Obstetrics and Gynecology
19. European Journal of Obstetrics & Gynecology and Reproductive Biology
20. Fertility and Sterility
21. Fertility Research and Practice
22. Fetal Diagnosis and Therapy
23. Ginekologia Polska
24. Gynecological Surgery
25. Gynecologic Oncology
26. Gynecologic Oncology Reports
27. Human Fertility
28. Human Reproduction
29. Human Reproduction Update
30. Hypertension in Pregnancy
31. International Journal of Fertility and Sterility
32. International Breastfeeding Journal
33. International Journal of Gynecology & Obstetrics
34. International Urogynecology Journal
35. Journal of Family Planning and Reproductive Health Care
36. Journal of Gynecologic Oncology
37. Journal of Lower Genital Tract Disease
38. Journal of Midwifery & Women's Health
39. Journal of Obstetrics & Gynaecology
40. Journal of Obstetrics and Gynaecology Canada
41. Journal of Obstetric, Gynecologic & Neonatal Nursing
42. Journal of Ovarian Research
43. Journal of Perinatal & Neonatal Nursing
44. Journal of Perinatal Medicine
45. Maternal Health, Neonatology, and Perinatology
46. Maturitas
47. MCN The American Journal of Maternal Child Nursing
48. Menopause Review (Przegląd Menopauzalny)
49. Menopause: The Journal of The North American Menopause Society
50. Neurourology and Urodynamics
51. Obstetrics & Gynecology
52. Paediatric and Perinatal Epidemiology
53. Placenta
54. Prenatal Diagnosis
55. Reproductive Health
56. The Breast Journal
57. The European Journal of Contraception and Reproductive Health Care
58. The Obstetrician & Gynaecologist (TOG)
59. Twin Research and Human Genetics
60. Ultrasound in Obstetrics & Gynecology
61. Women’s Midlife Health
